# Risk of liver dysfunction and non-alcoholic fatty liver diseases in people with hidradenitis suppurativa: A systematic review and meta-analysis of real-world evidences

**DOI:** 10.3389/fimmu.2022.959691

**Published:** 2022-12-14

**Authors:** Shuo-Yan Gau, Yu-Ping Hsiao, Wen-Chieh Liao, Kevin Sheng-Kai Ma, Meng-Che Wu

**Affiliations:** ^1^ School of Medicine, Chung Shan Medical University, Taichung, Taiwan; ^2^ Department of Medical Education, Chung Shan Medical University Hospital, Taichung, Taiwan; ^3^ Institute of Medicine, Chung Shan Medical University, Taichung, Taiwan; ^4^ Department of Dermatology, Chung Shan Medical University Hospital, Taichung, Taiwan; ^5^ Department of Post-Baccalaureate Medicine, College of Medicine, National Chung Hsing University, Taichung, Taiwan; ^6^ Department of Dermatology, Massachusetts General Hospital, Boston, MA, United States; ^7^ Department of Epidemiology, Harvard T.H. Chan School of Public Health, Boston, MA, United States; ^8^ Center for Global Health, Perelman School of Medicine, University of Pennsylvania, Philadelphia, PA, United States; ^9^ Division of Gastroenterology, Children’s Medical Center, Taichung Veterans General Hospital, Taichung, Taiwan

**Keywords:** hidradenitis suppurativa, liver diseases, non-alcoholic fatty liver disease, hepatitis B, hepatitis C

## Abstract

**Background:**

To date, evidences with high evidence-level evaluating the association between liver diseases and hidradenitis suppurativa was lacking. Given that inconsistency exists in some of the previous observational studies, evaluating the prevalence of liver diseases in HS patients could potentially serve as a reference of future guidelines for HS comorbidity screening. The aim of the current study was to evaluate potential association between hidradenitis suppurativa and liver diseases and provide integrated evidences.

**Methods:**

A search in PubMed, Web of Science and Embase based on the syntaxes ‘‘hidradenitis suppurativa’’ or ‘‘acne inversa’’ with “comorbidities”, “liver diseases”, “fatty liver” or “hepatitis” was performed. Observational studies evaluating epidemiological association between hidradenitis suppurativa and the risk of all liver diseases, including specific diseases as non-alcoholic fatty liver disease, hepatitis B, hepatitis C were targeted to be extracted in this systematic review and meta-analysis.

**Results:**

Within the initial 702 records, there were finally 8 real-world observational studies extracted. Results suggest that patients with HS are associated with all liver diseases (OR= 1.50; 95% CI, 1.27, 1.76), non-alcoholic fatty liver disease (OR= 1.78; 95% CI, 1.28, 2.48) and hepatitis B (OR=1.48; 95% CI, 1.12, 1.94), but not hepatitis C (OR= 1.27; 95% CI, 0.78, 2.07). HS patients were associated with significantly increased risk of liver diseases, especially the risk of non-alcoholic fatty liver disease and hepatitis B.

**Conclusions:**

Clinicians should be alert to the clinical relationship while caring people with hidradenitis suppurativa and the screening of liver function should be recommended to HS patients.

**Systematic review registration:**

https://www.crd.york.ac.uk/prospero/, identifier CRD42022296034.

## Highlights

- Current meta-analysis suggested HS patients have higher risk of all liver diseases, non-alcoholic fatty liver disease and hepatitis B, but not hepatitis C
- Liver function screening should be recommended to HS patients for comorbidity screening in future guidelines

## Introduction

As a chronic disorder associated with many immunological comorbidities, hidradenitis suppurativa (HS) causes impairment to patients’ quality of life (1). The prevalence of HS was estimated to be 0.4% in Western countries (2). Comorbidities of HS including dermatological, cardiovascular and endocrinological events (3). Though the actual immunologic mechanism of HS has not be fully developed, it was reported that HS was involved in the increased secretion of inflammatory cytokines including IL-17 and TNF alpha (4).

Liver dysfunctions were thought to be involved in the pathophysiology of chronic inflammatory dermatologic diseases (5, 6). Presence of inflammatory skin diseases such as psoriasis and bullous pemphigoid were thought to influence the pathogenesis of viral hepatitis (7, 8). The interaction between non-alcoholic fatty liver diseases (NAFLD) and other inflammatory dermatological events has also long been discussed (5). Mechanisms including the increase of proinflammatory cytokines and related metabolic syndromes could play a role in the interaction between inflammatory skin diseases and liver diseases such as NAFLD and viral hepatitis (9–11).

Chronic inflammatory status could potentially trigger subsequent endocrinological events, leading to multi-systemic consequences (12–15). However, to date, evidences with high evidence-level evaluating the association between liver diseases and HS was lacking. A recent guideline indicated that since association between HS and non-alcoholic fatty liver disease (NAFLD) was unsure due to insufficient evidences, NAFLD screening in HS patients could not be recommended for comorbidity screening in HS pateints (3). Hence, it’s necessary to clarify the association between HS and NAFLD. Additionally, aside from NAFLD, other liver diseases were also reported to have high prevalence in HS patients (16, 17). Given that inconsistency exists in some of the results, evaluating the prevalence of liver diseases in HS patients could be practical and could potentially serve as a reference of future guidelines for HS comorbidity screening. Hereby, we conducted a systematic review and meta-analysis to evaluate potential association between HS and liver diseases and provide integrated evidences.

## Methods

### Evidence search, screening process and eligibility criteria

The current study abided by the Preferred Reporting Items for Systematic Reviews and Meta-analyses (PRISMA) (18). Observational studies evaluating epidemiological association between HS and the risk of all liver diseases, including specific diseases as NAFLD, hepatitis B, hepatitis C were targeted to be extracted in this systematic review and meta-analysis. This study project has been registered in PROSPERO with an ID number of CRD42022296034.

On December 12, 2021, we performed a search in PubMed, Web of Science and Embase based on the syntaxes ‘‘hidradenitis suppurativa’’ or ‘‘acne inversa’’ with “comorbidities”, “liver diseases”, “fatty liver” or “hepatitis”. In the searching process, characteristics of studies such as language or ethnics of study population was not limited. Searching protocol and detailed information of PICO (participants, intervention, comparison and outcome) could be found in the **Supplementary files**. Similar study design and study extraction criteria has been applied in published studies (19, 20).

Observational studies, including cohort studies, case-control studies and cross-sectional studies were regarded as having eligible study designs for extraction. Studies would be excluded in the screening process if meeting any of the following criteria: (1) Unrelated to HS (2) HS related but not evaluating comorbidity association (3) HS comorbidity related but not mentioning any HS-liver disease association (4) *in vivo*/*in vitro* studies or genetic studies (5) HS-liver disease related but not having appropriate comparative arms. To address publication bias, conference abstracts were not excluded.

### Data extraction and study quality assessment

After screening of studies, information regarding authors’ name, study design, year each study published, location each study conducted, study outcome of interest, definition of HS patients and outcome events, gender percentage, mean age and the number of participants were extracted from the studies meeting our eligibility criteria. Extracted characteristics were recorded in **Table 1**. Newcastle-Ottawa Scale was applied to evaluate study quality (21).

**Table 1:** Characteristics of included studies.

Author	study design	Year	Location	ParticipantSource	Study Outcome	definition of HS patient	definition of outcome event	female percentage case/control (%)	mean age (yo) case/control	No. of participants
Lee et al	cross sectional	2018	Korea	Korean NHI claims database	autoimmune hepatitis	at least two documented physician contacts based on ICD-10 code L729	at least 2 physician diagnoses based on ICD-10 code K754	38.7/38.7	33.6/33.6	171096
	cohort (moderate HS)					at least two diagnoses for ICD-9-CM 705.83	medical claims recorded	74/74	42.19/42.19	4584
Kimball et al	cohort (severe HS)	2018	US	OptumHealth Care Solutions, Inc. database	liver diseases	at least two diagnoses for ICD-9-CM 705.84 and experienced at least one of the disease severity indicators	medical claims recorded	71/71	42.19/42.19	6130
Durán-Vian et al	case-control	2019	Spain	Recruited patients from University Hospital Marqués de Valdecilla (Santander, Spain)	NAFLD	HS diagnostic criteria	sonographic characteristics	35/86	44.4/46.2	220
Narla et al	cross sectional	2019	US	National Inpatient Sample	nonalcoholic steatohepatitis	NA	NA	NA	NA	NA
González-Villanueva et al	cross sectional	2020	Spain	Patients from HS clinic of Alicante Institute for Health and Biomedical Research (ISABIAL-FISABIO Foundation; Alicante, Spain)	NAFLD	Assessed by dermatologist based on clinical exams and findings	sonographic characteristics	64/62	47/49	245
Lee et al	cross sectional	2020	US	National Inpatient Sample	Hepatitis A, B, C	Diagnoses based on ICD-9-CM 705.83	Diagnoses based on ICD-9-CM codes	60.3/58.6	41.33/47.89	87053155
Raiker et al	cross sectional	2020	US	National Inpatient Sample	NAFLD	NA	NA	NA	NA	NA
Cohen et al	cross section	2021	Israel	Clalit Health Services (CHS) database	Hepatitis B, C	at least twice by outpatient physicians or once in hospital discharge documentation	at least twice by outpatient physicians’ diagnosis or once in hospital discharge documentation	62.6/62.6	38.6/38.6	26502

HS, Hidradenitis suppurativa; NAFLD, non-alcoholic fatty liver disease; ICD-9, International Classification of Diseases, 9th Revision; ICD-10, International Classification of Diseases, 10th Revision; y/o, years old; NA, not available.

### Statistical analysis

To perform qualitative synthesis, pooled odds ratio was calculated based on the data extracted from eligible observational studies. In all analysis models in the current study, random effect model was applied to address possible clinical heterogeneity. To evaluate the heterogeneity within pooled studies, value of I (2) was applied and would be presented in each forest plot. With a I (2) value greater than 75%, heterogeneity could be considerable (22). For the presentation of odds ratio, 95% confidence interval (95% CI) were applied. Review Manager 5.4 (Cochrane, London, UK) were utilized for statistical analyses.

## Results

### Characteristics of included studies

**Figure 1:**
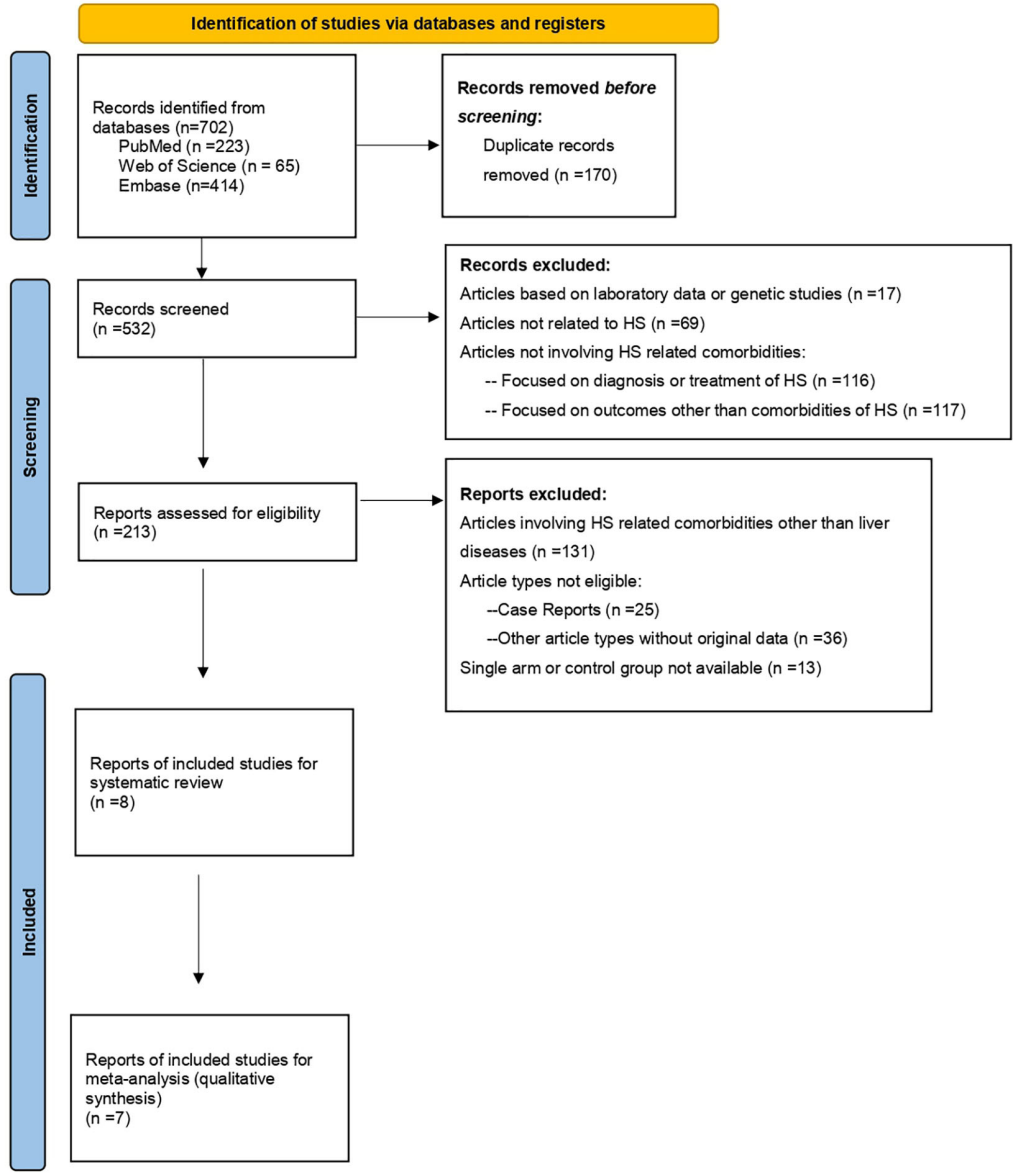
PRISMA Study Flowchart (18).

As presented in **Figure 1**, within the initial 702 records, there were finally 8 real-world observational studies [1 cohort study (23), 1 case-control study (24) and 6 cross-sectional studies (1, 16, 17, 25–27)] included for data extraction after screening process. Most of the included studies were conducted in North America and Europe [4 studies conducted in United States (16, 23, 26, 27) and 2 in Spain (24, 25)], whereas one-third of the studies were conducted in Asia [1 study conducted in Korea (23) and 1 in Israel (1)]. Within the included studies, outcomes including NAFLD, hepatitis B, hepatitis C and all liver diseases had been extracted (**Table 1**).

### Quality evaluation of included studies

Within included studies risk of bias had been assessed by Newcastle-Ottawa Scale. (**Figures S1A, B**). Four cross-sectional studies (16, 17, 26, 27) had been evaluated to have unknown bias regarding the adequacy of case definition since dermatologist of specialists were not reported to be involved in the identification of cases. For case identification and diagnosis, provided information was insufficient or only the International Classification of Diseases, 9th Revision/10th Revision (ICD-9/10) was applied as the identification criteria. Two studies were evaluated to have unknown bias to non-response rate and same methods ascertaining controls and cases due to insufficient information. Publication bias could exist in the extracted studies due to asymmetric results in the funnel plot

### Qualitative synthesis: HS-liver disease association

#### HS-All pooled liver diseases and hepatitis (hepatitis B; hepatitis C)

**Figure 2:**
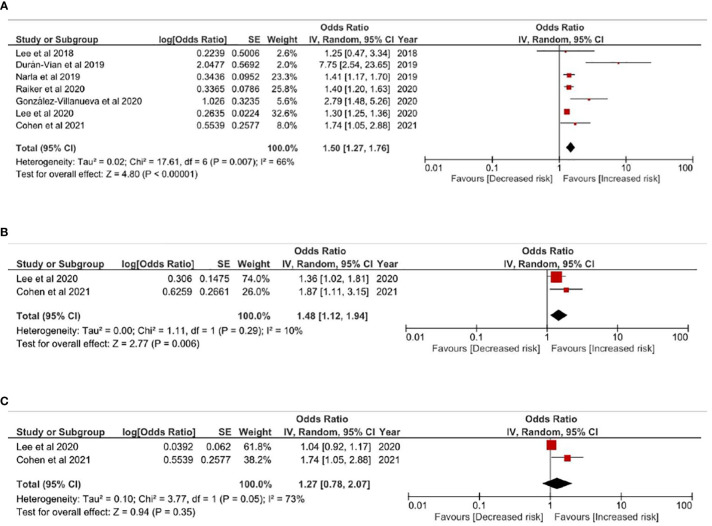
**(A)** Odds ratio of all pooled evidences of liver diseases in people with Hidradenitis Suppurativa **(B)** Odds ratio of hepatitis B in people with hidradenitis suppurativa **(C)** Odds ratio of hepatitis C in people with hidradenitis suppurativa.

Comparing with non-HS people, patients with HS were associated with higher risk of liver diseases (OR=1.50; 95% confidence interval, 1.27-1.76), with moderate heterogeneity between pooled studies (I^2 =^ 66%) (**Figure 2A**). validate the HS-liver disease association. Given that representative of a population could be different between baseline of case-control studies and cross-sectional studies, a sensitivity model has been performed based on including cross-sectional studies in the analysis. The association remained statistically significant in the sensitivity analysis (**Figure S2**). Subgroup analyses was performed to evaluate risk of viral hepatitis in HS people (**Figures 2B, C**). Based on studies with low to moderate heterogeneity, HS patients had statistically significant risk for having hepatitis B. (OR=1.48, 95% CI, 1.12-1.94). For hepatitis C, the association was not statistically significant (p=.35).

#### HS-NAFLD

**Figure 3:**
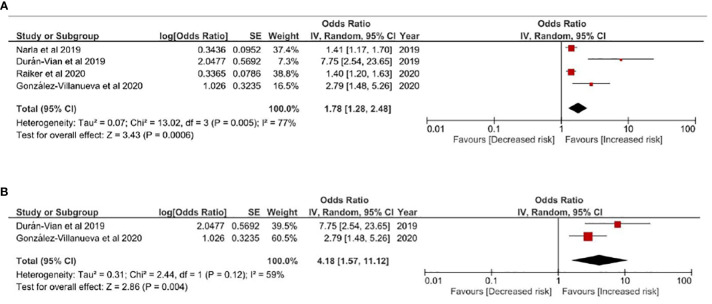
**(A)** Odds ratio of all pooled evidences of NAFLD in people with Hidradenitis Suppurativa **(B)** Sensitivity Analysis: Odds ratio of NAFLD in people with Hidradenitis Suppurativa based on results controlling obesity related factors Legends: Given obesity was highly involved in both HS and NAFLD, obesity-related factors were critical of considering while evaluating HS-NAFLD association. In this model, studies not adjusting obesity related factors such as BMI or metabolic syndrome comorbidities were excluded from the qualitative synthesis.

In **Figures 3A, B**, risk of NAFLD in HS patients was evaluated. HS patients were associated with higher risk of NAFLD comparing with non-HS controls, with a pooled odds ratio of 1.78 (95% CI, 1.28-2.48) (**Figure 3A**). Given obesity was highly involved in both HS and NAFLD, obesity-related factors were critical of considering while evaluating HS-NAFLD association. Thereby, we performed a sensitivity analysis based on an additional model only including studies adjusting obesity related factors such as BMI or comorbidities related to metabolic syndrome in the qualitative synthesis. In this model, a 4.18-fold risk of having NAFLD was observed in people with HS (95% CI, 1.57-11.12) (**Figure 3B**). The observed association was validated in another sensitivity analysis including only cross-sectional studies (**Figure S3**). In all models evaluating HS-NALFD association, moderate heterogeneity was showed within included studies.

## Discussion

The present systematic review and meta-analysis provides integrated evidences of the increased risk of liver diseases, including NAFLD and hepatitis B in people with HS. To the best of our knowledge, this is the first study providing pooled odds ratio regarding the HS-liver disease association. Given that the current recommendation for HS comorbidity screening did not provide clear information regarding the liver comorbidities of HS (3), the results provided in this study could potentially serve as a critical reference of future guidelines for HS comorbidity screening.

Being viewed as a disease involving multiple organic system and highly associated with metabolic system, the term “Metabolic-associated Fatty Liver Disease (MAFLD)” was designed to clinically describe NAFLD (28). NAFLD and MAFLD were reported to share similar clinical status and long-term outcomes (29). Whichever term utilized, the status of fatty liver disease was thought to be massively influenced by the status of obesity and metabolic syndrome. Therefore, in studies evaluating NAFLD-related association, obesity and comorbidities status could potentially cause great confounding bias. In the current study, the sensitivity analysis considering BMI and metabolic-syndrome-related comorbidities information could serve as a validation of the HS-NAFLD association. In the obesity-adjusted model the risk was even higher than other models, presenting a more than 4-fold risk. Though current evidence was validated in three different sensitivity models, the analysis was limited to insufficient data, for only two studies was available for obesity adjustment (24, 25). Future eligible observational evidences considering the influence of obesity were necessary to make the integrated evidence more rigorous. However, the current three models could provide a critical reference regarding the significantly increased risk of NAFLD in HS patients.

The reported association between HS and liver diseases could potentially attributed to elevated proinflammatory cytokines and adipokines involved in the chronic inflammatory status of HS.

**Figure 4:**
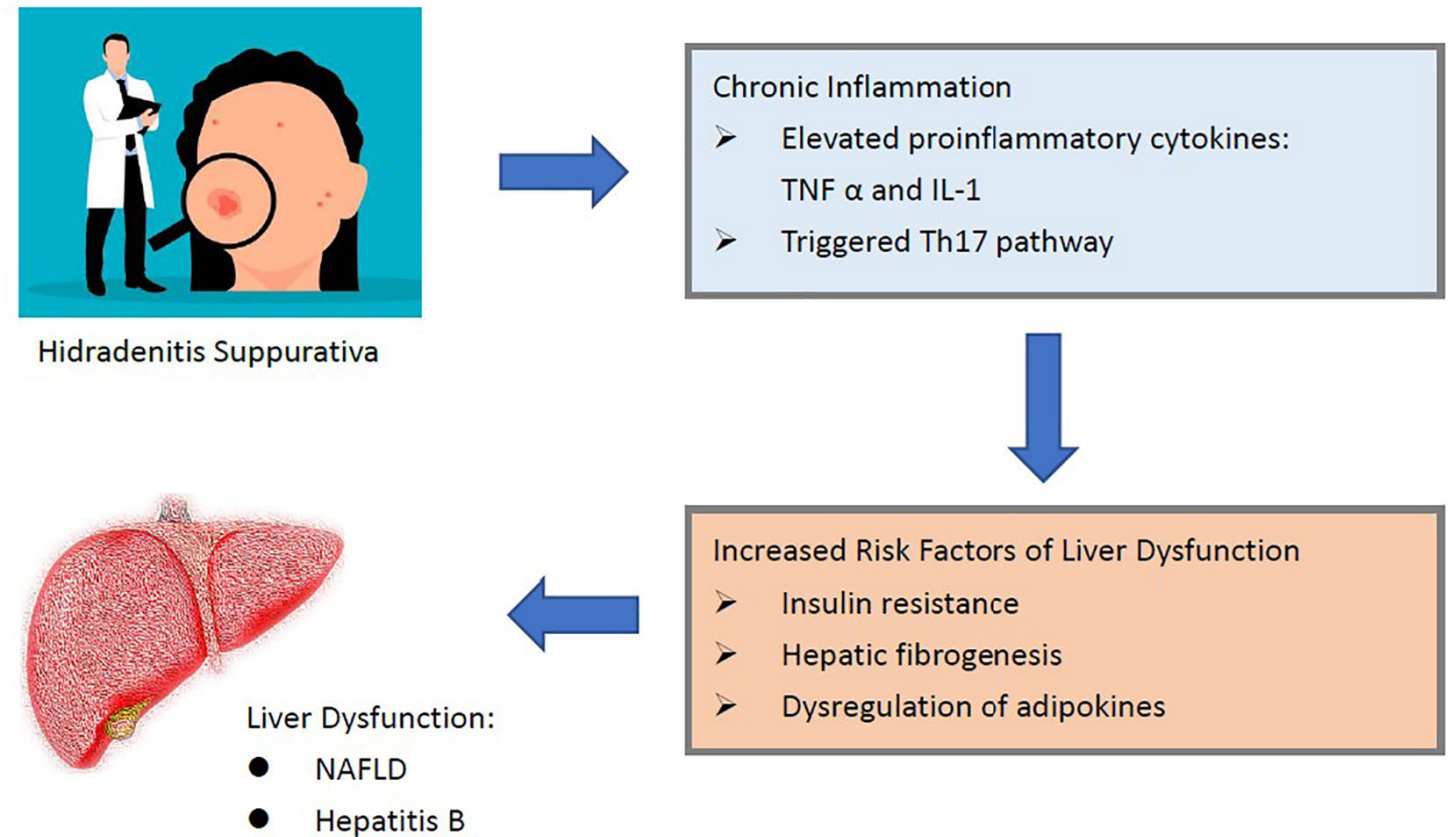
Potential mechanisms between HS and liver diseases.

In the pathogenesis of inflammatory skin diseases, elevated cytokines such as tumor necrosis factor alpha (TNF alpha) or interleukin 1 (IL1) could potentially lead to impairment in liver function (5, 24, 25). Immunological links between inflammatory skin diseases, for instance, psoriasis, could also be potentially attributed to the role of low-grade chronic inflammation. In psoriasis patients, presence of chronic inflammation could lead to the elevation of TNF alpha, which could in turn increase the risk of insulin resistance and hepatic fibrogenesis (30). These consequences could serve as risk factors of NAFLD (30) (**Figure 4**). Additionally, recent real-world evidences also indicated that HS and psoriasis could have bidirectional association (5). Current studies had been finding the actual inflammatory pathway involved in the pathogenesis of HS. Though the full theory of HS-caused inflammation has not been well-developed, it was reported that Th17 family also played a great role in the HS inflammation pathway (11, 31). Medications against IL-17 were thought to be potential choice for HS treatments (32, 33). As for the development of NAFLD, mechanisms related to Th17 were also highly involved (10, 34). In viral hepatitis, high level of IL17 was also presented in people with HBV and HCV infection and could be associated with severer disease status (9, 35). In previous studies, dysregulation of serum Th17 cells and IL17 concentration were observed in patients with hepatitis B. Given that hepatitis B and HS were involved in common immunological pathways, the high prevalence could possibly be explained. Moreover, in people with HS, status of adipokines, including adiponectin, resistin and leptin were reported to be dysregulated (36). In the pathogenies of NAFLD, imbalanced adipokines were also thought to play a potential role (37, 38). Though common mechanisms in immunological pathways existed, actual mechanisms have not been completed developed to determine the HS-liver diseases relation. Further lab-based studies were needed to clarify the interaction between inflammatory system, cytokines, adipokines and liver function in HS patients.

## Limitations

The current study was limited to current available data. First, given that available observational data was insufficient to perform stratification analysis of age or gender, we were not able to evaluate whether age and gender could show difference on the HS-liver diseases association. Second, since in most of the included databased-based retrospective studies, information regarding severity indicators of HS such Hurley Stage were not available, we were not able to consider the severity of HS as common covariate in the pooled model. Third, for the identification of NAFLD, most studies did not clarify the difference between non-alcoholic steatohepatitis and non-alcoholic fatty liver, for the two subgroups of NAFLD referred to different disease severity. Third, in most studies, alcoholism status was not provided, which could lead to potential confounding bias. Lacking information regarding alcohol uptake could be a potential limitation in both studies evaluating HS-NAFLD association and comprehensive analyses based on these real-world studies. Fourth, the current study is limited due to the small amount of eligible evidences, and the extracted studies were less than 10 studies. However, it is not recommended to present publication bias *via* funnel plot when studies were less than 10 due to low evidence power (22).Readers should be cautious while interpreting the reported results of the current study.

## Conclusion

As a conclusion, we report a significantly increased risk of liver diseases in HS patients, especially the risk of NAFLD and hepatitis B. Clinicians should be alert to the clinical relationship while caring people with HS. Future studies should focus on whether the difference between HS severity and duration would affect the extent of the observed association.

## Data availability statement

The original contributions presented in the study are included in the article/**Supplementary Material**. Further inquiries can be directed to the corresponding author.

## Author contributions

All the authors involved in drafting or revising the article and approved of the submitted version. Study conception and design: S-YG, Y-PH, KM and M-CW. Data analysis and demonstration: S-YG and M-CW. Original draft preparation: S-YG and M-CW. All authors contributed to the article and approved the submitted version.
