# Supplementary material for: Risk of liver dysfunction and non-alcoholic fatty liver diseases in people with hidradenitis suppurativa: A systematic review and meta-analysis of real-world evidences
Source: Front Immunol. 2022 Dec 14;13:959691. doi: 10.3389/fimmu.2022.959691 (PMC9794989; doi:10.3389/fimmu.2022.959691)
Supplement: Supplementary file 1 [file DataSheet_1.pdf]

Supplementary Files:

1. **Supplementary Statements**

   PICO and study inclusion/exclusion criteria:

   Participants:

   All patient with hidradenitis suppurativa (age, sex and other baseline characteristics were not limited)

   Intervention: NA

   Comparison:

   All people without hidradenitis suppurativa (age, sex and other baseline characteristics were not limited)

   Outcomes:

   Liver diseases, subgroups including non-alcoholic fatty liver diseases, hepatitis B and hepatitis C

   Inclusion and exclusion criteria:

   Only studies meeting the PICO will be included. Articles not evaluating the prevalence of liver diseases in hidradenitis suppurativa and those without appropriate control group will be excluded from data extraction. For meta-analysis, only studies with similar adjustment model and study design will be pooled.

Searching syntaxes: Retrieved on 2021.12.12

Embase, n=414

(suppurative AND hidradenitis OR (acne AND inversa) OR (hidradenitis AND suppurativa)) AND (liver AND disease OR hepatitis OR (fatty AND liver) OR comorbidity) NOT review

Web of Science：n=65

((ALL=(hidradenitis suppurativa) OR (ALL=(acne inversa))AND ((ALL=(liver disease))OR (ALL=(hepatitis)) OR (ALL=(fatty liver))OR (ALL=(comorbidity)))) NOT ALL=(Review)

Pubmed, n=223

(hidradenitis suppurativa OR acne inversa) AND (liver disease OR hepatitis OR fatty liver OR comorbidity) NOT review

2. Supplementary Figures

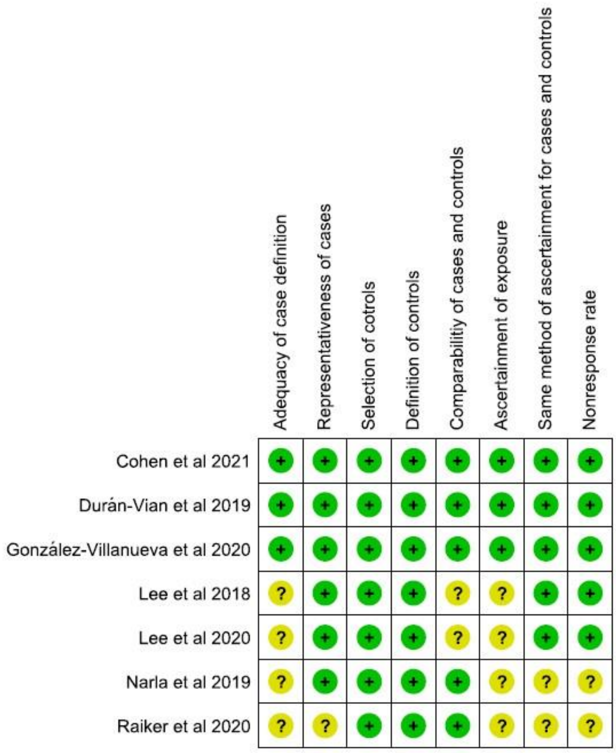

Figure S1(a). Risk of bias assessment for cross-sectional and case-conrtol studies

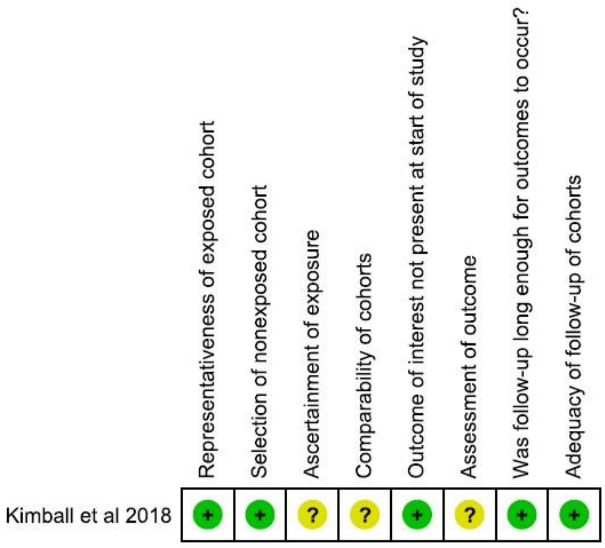

Figure S1(b). Risk of bias assessment for cohort studies

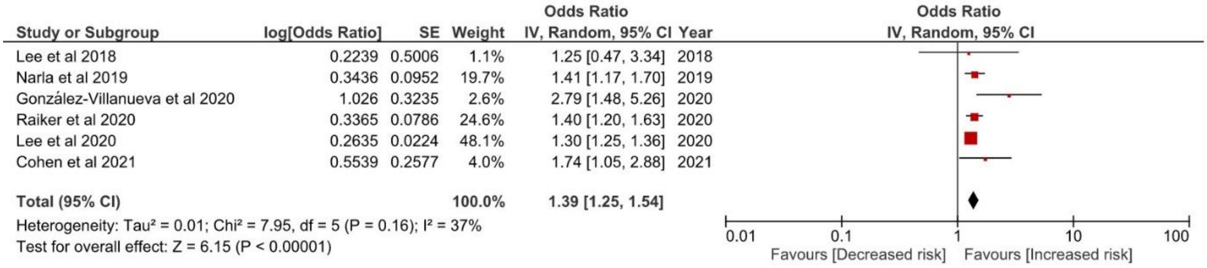

Figure S2. Sensitivity Analysis: Odds ratio of liver diseases in people with Hidradenitis Suppurativa

Legends: This model was designed to validate the HS-liver disease association. Representative of a population could be different between baseline of case-control studies and cross-sectional studies. Hence, the case-control study by **Durán-Vian et al** was not included in this sensitivity analysis since it was different from other included studies in this analysis.

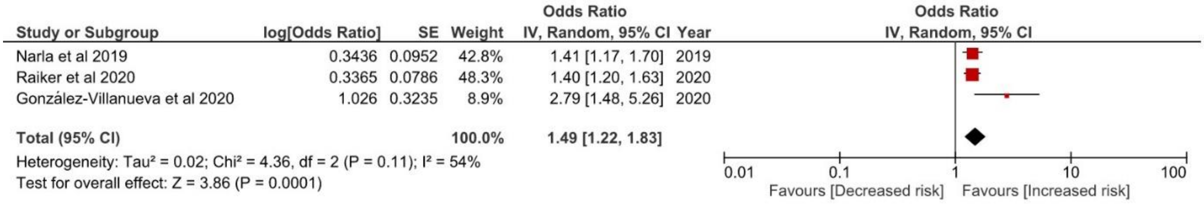

Figure S3. Sensitivity Analysis: Odds ratio of NAFLD in people with Hidradenitis Suppurativa

Legends: This model was designed to validate the HS-NAFLD association. Representative of a population could be different between baseline of case-control studies and cross-sectional studies. Hence, the case-control study by **Durán-Vian et al** was not included in this sensitivity analysis since it was different from other included studies in this analysis.
